# Treatment of benign biliary strictures with expandable biodegradable stents: Safety and efficacy in a single center

**DOI:** 10.1055/a-2813-3346

**Published:** 2026-02-27

**Authors:** Gabriel Marcellier, Abdellah Hedjoudje, Benedicte Jais, Frederique Maire, Kenza Bourhrara, Alain Berson, Fabiano Perdigao, Olivier Scatton, Heithem Soliman, Paul Rivallin, Frédéric Prat

**Affiliations:** 1 55100 Endoscopy Unit, Beaujon Hospital, Clichy, France; 2 372959 Endoscopy Unit, Ambroise Pare Hospital, Neuilly-sur-Seine, France; 3 55100 Pancreatology, Beaujon Hospital, Clichy, France; 4 Hepatobiliary Surgery, Pitié-Salpêtrière Hospital, Paris, France; 5 26931 Heptobiliary Surgery, Hospital Louis-Mourier, Colombes, France

**Keywords:** Pancreatobiliary (ERCP/PTCD), Strictures, Endoscopic ultrasonography, Biliary tract, Quality and logistical aspects, Performance and complications

## Abstract

**Background and study aims:**

Benign biliary strictures (BBS) are commonly managed by progressive calibration using plastic or metallic stents. Although fully-covered metallic stents (FC-SEMS) enable immediate calibration to a larger diameter compared with plastic stents, they remain prone to migration and use is limited in intrahepatic and peri-hilar strictures. We report on using uncovered expandable bioresorbable stents (BRES) in a series of selected BBS patients.

**Patients and methods:**

This retrospective monocentric case series included all consecutive patients treated between 2023 and 2024. Patients were highly selected for uncommon situations for which usual stents were not well suited and followed for at least 12 months after the procedure. Technical success, clinical success, and adverse events (AEs) were systematically recorded.

**Results:**

Five procedures were performed in five patients with implantation of a total of eight UNITY-B stents. Three patients underwent internalization of an internal-external drainage across a bilio-digestive anastomotic stricture. One patient was treated with retrograde extra-anatomical endoscopic drainage for an anastomotic stricture. One patient underwent calibration of an intrahepatic stricture following radiofrequency of an IPMN-B. Technical success was achieved in all cases (100%), with clinical success observed in 80% of patients. No AEs were observed.

**Conclusions:**

Use of bioresorbable UNITY-B stents appears feasible and safe for selected benign biliary strictures, including in intrahepatic locations. Further studies are needed to confirm these preliminary findings.

## Introduction

Benign biliary strictures (BBS), postsurgical or otherwise, can be difficult to manage 1 . Endoscopic retrograde cholangiopancreatography (ERCP) remains the main therapeutic option with dilation and stenting of the stricture 2 . European guidelines recommend against use of uncovered self-expandable metallic stents (UC-SEMSs) in benign strictures because they cannot be easily removed 3 . For benign strictures of the main biliary duct, such as post liver-transplant anastomosis, recommendations tend to underscore the superiority of fully covered SEMS (FCSEMSs) over multiple plastic stents (MPSs) in terms of number of procedures needed to achieve remission, although an additional procedure remains mandatory for stent removal 4 5 . However, if the biliary stricture is intrahepatic or too close to the hilum, risk of excluding accessory bile ducts precludes using FCSEMSs, leaving no other option than iterative calibration every 3 to 4 months with MPS. Moreover, a common limitation of FCSEMSs as well as plastic stents is their migration rate of up to 24% in benign indications 6 . When biliary surgery does not allow using ERCP to access the biliary tract, a percutaneous approach is often required involving placement of a protracted percutaneous access (external or internal-external drain) due to absence of internal biliary access for retrieval.

A stent combining the advantages of an uncovered design (low migration risk, possibility of intrahepatic positioning with lateral patency that does not obstruct biliary side branches) bioresorbability, and no requirement for endoscopic removal after a predefined calibration period would represent a significant advance in management of BBSs.

Because an innovative hybrid platform of expandable magnesium and polymer stent has been recently made available, we report in this case series on our early experience with this device.

## Patients and methods

**Fig. 1:**
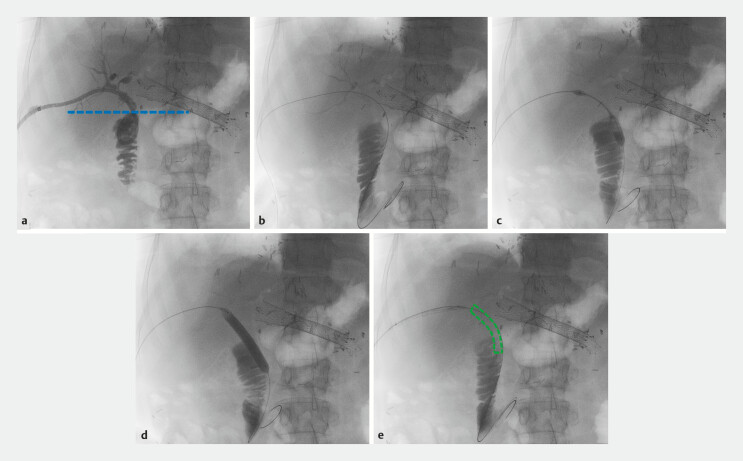
Illustration of Case 3, percutaneous internalization of an internal-external drain with a UNITY-B bioresorbable stent. **a** Opacification through the internal-external drain. The blue line represents the anastomosis. **b** Replacement of the drain with a guidewire. **c** Dilation of the balloon contained into the bioresorbable stent. **d** Full opening of the balloon allowing deployment of the stent at a diameter of 10 mm. **e** Removal of the balloon, the stent is poorly visible and is highlighted in green.

From March 2023 to December 2024, highly selected patients with BBS were treated using UNITY-B (QualiMed, Q3 Medical group, Winsen, Germany and Charlotte, North Carolina, United States) bioresorbable and expandable stents (BRESs). BRESs were positioned by a single expert endoscopist (FP) at a tertiary referral academic center (Beaujon Hospital, Clichy, France). Positioning could be either percutaneous, if an external or internal-external drain had been previously inserted by radiologists, or endoscopic during ERCP because the stent is available with either a short or a long delivery system with an 8F catheter. UNITY-B stents are CE-approved and made of a magnesium-based polymer alloy. Contrary to nitinol-based FCSEMS, these stents do not exert radial expansion force when unloaded, but must be balloon-inflated using the built-in balloon up to 8 or 10 mm depending on the desired diameter. The stents have an uncovered design and the mesh is impacted in the bile duct wall to maintain the specified diameter. When the balloon is deflated and removed, the stents remain in place. Radiopaque markers on both ends of the balloon help position the stents but it is noteworthy that the stents themselves are very poorly visible on either computed tomography (CT) scan or fluoroscopy once deployed and the balloon is removed ( Fig. 1 ). The uncovered design allows intrahepatic placement of the stents without covering collateral bile ducts and prevents migration. Stents used in this case study have a theoretical (lab-studied) dissolution period of 3 months. We used 5.7 cm-long stents, meaning a couple of overlapping stents was an option to adjust for longer strictures.

Information about patients and clinical outcome data were collected prospectively but the histories, stenting indications, procedure details, and overall outcomes were analyzed retrospectively by two senior endoscopists (GM, PR). Data were collected after ensuring patient non-opposition. No Institutional Review Board approval was required in this retrospectively designed study.

Our main outcome was clinical success, defined by biliary patency at 12 months from BRES stenting. Secondary outcomes included technical success, defined by the ability to properly position the stent during the procedure, adverse events (AEs), graded following the Clavien-Dindo classification 7 , defined as any procedure-related events occurring during the first 30 days following stenting, and relapse rates, defined as loss of biliary patency at the last follow-up available. Times of last follow up were reported in a timeline plot and relapse-free times illustrated through a Kaplan-Meier curve.

After BRES stenting, patients were closely followed with clinical and biological examinations. Recurrence was suspected in case of clinical manifestations (cholangitis, pruritus) or in the presence of abnormal liver function tests (LFTs). Suspected loss of biliary patency was confirmed by imaging (CT scan or magnetic resonance imaging). Accordingly, a symptom-free patient with significant improvement in LFTs (< 1.5 × the upper limit of normal or ≥ 50% reduction compared with pre-stent levels) 12 months after BRES was considered indicative of stricture resolution.

## Results

Five BRES procedures among five patients including eight stent positionings were collected in this case series. Median age was 64 years [55, 56, 57, 58, 59, 60, 61, 62, 63, 64, 65, 66, 67, 68]. Two patients were male and three female. All patients had non-tumoral biliary strictures. Four had an altered anatomy with a history of Roux-en-Y reconstruction and a biliary anastomotic stricture. For three of them, percutaneous internal-external drainage had been established before BRES stenting. In another patient, an EUS-guided endoscopic choledocojejunostomy was done by using a FCSEMS, followed by anterograde BRES stenting of the neoanastomosis during a subsequent procedure. In those four cases, BRES stenting allowed calibration of the anastomosis to a diameter of up to 8 to 10 mm, permitting removal of the percutaneous drain in the first three cases and of the FCSEMS in the fourth case.

One patient had a benign but pre-neoplastic stricture of the left hepatic duct related to intraductal papillary mucinous neoplasm of the bile duct (IPMN-B), as confirmed by retrograde cholangioscopy. The patient had refused the proposed left lobectomy but accepted a conservative option. After total ablation using fluoroscopy-guided radiofrequency (Endo-HPB, Boston Scientific, United States), bioresorbable stenting allowed calibration of the intrahepatic stricture at up to 10 mm while preventing occlusion of accessory bile ducts and allowing for larger calibration than what plastic stents would have provided.

Technical success was achieved in 100% of cases. Clinical follow-up ranged from 12 to 31 months. Twelve-month clinical success was achieved in 80% of patients with one patient experiencing a global failure of the stenting with early relapse of the stricture, implying another internal-external drainage. Follow-up revealed tumor relapse, explaining the early stricture unfavorable outcome, and leading to patient death 18 months after the BRES procedure.

Two patients had complete clinical success with complete calibration of their stricture with normal LFTs and no cholangitis or need for reintervention at 20 and 24 months from the intervention, respectively. One patient developed intrahepatic segmentary dilations, possibly related to a relapse of her initial cholangitis with liver function that never completely normalized. However, she had successful calibration of her biliodigestive anastomosis without any need for reintervention and was considered as a clinical success. The last patient with an IPMN-B-related intrahepatic stricture treated with intraductal radiofrequency ablation (RFA) and BRES had control cholangioscopy performed at 6 months post-procedure that showed no residual tumor or neoplastic stigma, no remaining stent debris or stone formation, and no significant stricture of the left hepatic duct, although hydrostatic dilation at 4 mm was needed to pass the 10F cholangioscope beyond the treated segment but without need of additional stenting. At 12 months after original stenting and RFA, a second control was performed without degradation of LFTs or relapse of the stricture at cholangioscopy.

**Fig. 2:**
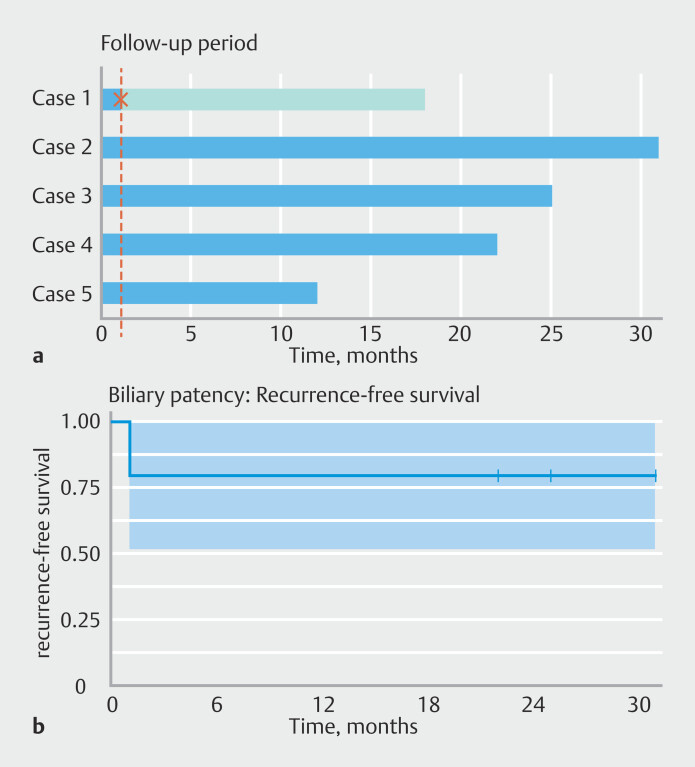
Timeline plot ( **a** ) illustrating the follow-up period for each patient after BRES stenting. Each horizontal bar represents one patient (blue in the absence of recurrence and orange if recurrence happened). The cross marks the recurrence time of the biliary obstruction. Kaplan-Meier ( **b** ) curves illustrating the biliary patency survival over follow-up.

Table 1 highlights patient characteristics as well as procedure details, outcomes, and complications. Fig. 1 illustrates the case of Patient 3 with replacement of an internal-external drain with a UNITY-B stent. Fig. 2 represents the timeline plot illustrating the follow-up period for each patient. Regarding safety, there were no AEs related to the procedure in any of the cases.

**Table:** **Table 1** Clinical cases: Description, procedural details, outcome, and adverse events.

**Patient characteristics and indication**	**Procedure description**	**Outcomes**	**AEs**
**Case 1** 64-year-old male Pancreatico-duodenectomy for an ampullary adenocarcinoma Stricture of the biliodigestive anastomosis Percutaneous transhepatic drainage Bioresorbable stent for internalization of drainage	Contrast in the internal-external radiological drain confirmed trans-anastomotic positioning Removal and exchange with a guidewire. Opacification highlighting a hilar stricture Positioning of a first 5.7 cm x 10 mm BRES through the anastomosis Positioning of a second one creating a bridge between left and right liver to prevent exclusion of the left liver by the stent’s opening	**Technical Outcome:** *Success* Successful percutaneous insertion of two bioresorbable stents under fluoroscopy allowing removal of drain **Clinical Outcome:** *Unsuccessful* Early relapse of the stricture with increased LFTs and bile duct dilation at 1 month on CT scan. Repeat internal-external drainage one month after BRES stenting with positioning of a single uncovered SEMS. No visibility of the stents on CT scan at 1 month (As the stents are poorly visible, it was impossible to conclude between early dissolution, migration or obstruction of the stent) **Follow-Up** (18 months): *Relapse* Tumoral relapse confirmed with afferent loop syndrome requiring an endoscopic gastro-jejunostomy. Patient deceased 18 months after initial procedure from tumor progression.	No procedure-related AEs
**Case 2** 68-year-old male Pancreatico-duodenectomy for malignant IPMN. Stricture of the biliodigestive anastomosis. Failure of endoscopic hepaticogastrostomy but previous successful EUS-guided choledoco-jejunostomy stented with a FCSEMS. Persistence of anastomotic stricture with choledocholithiasis.	Positioning in the jejunum in front of the FCSEMS allowed access to the common bile duct. Persistence of a biliary stone above the anastomotic stricture. Removal of the FCSEMS and catheterization through the orifice with the wire crossing the anastomotic stricture. Positioning of two coaxial BRES of 5.7 cm and 10 mm covering the anastomotic stricture and calibrating the common bile duct until the hilum.	**Technical Outcome:** S *uccess* Successful endoscopic insertion of two coaxial bioresorbable stents calibrating the anastomotic stricture **Clinical Outcome** : *Success* Early normalization of LFTs after the procedure, sustained at 6 and 12 months. **Follow-Up** (31 months): *No relapse* Disappearance of the choledocholithiasis and no remaining stricture or bile duct dilation with persistent hemobilia confirmed by follow-up EUS 2 years after stenting. Persistent pancreatic duct dilation with IPMN relapse on the pancreatic remnant.	No procedure-related AEs
**Case 3** 55-year-old female Left hepatectomy for large hemangioma followed by liver transplantation with biliodigestive anastomosis. Portal thrombosis with a cavernoma followed by portal cholangiopathy inducing a stricture of the biliodigestive anastomosis 2 years after transplantation. Percutaneous transhepatic drainage in place. BRES for internalization of drainage.	Contrast in the internal-external radiological drain confirmed their trans-anastomotic positioning. Removal and exchange with a guidewire. Positioning of a 5.7-cm BRES (dilated at 8 mm) through the anastomosis	**Technical outcome** : *Success* Successful percutaneous insertion of a bioresorbable stent through the anastomotic stricture and removal of the internal-external drain **Clinical outcome** : *Success* Normalization of LFTs sustained at 12 months. **Follow-up** (25 months): *No relapse* No clinically-suspected relapse.	No procedure-related AEs
**Case 4** 37-year-old female Liver transplantation 12 years ago for primary sclerosing cholangitis. Right split liver with separate biliodigestive anastomoses for anterior and posterior bile ducts Strictures of both anastomoses drained percutaneously. BRES for internalization of drainage.	Contrast in both internal-external radiological drains confirmed their trans-anastomotic positioning Removal of the drains and exchange with guidewires. Positioning of two 5.7-cm x 10-mm BRES, one through each anastomosis	**Technical outcome** : *Success* Successful percutaneous insertion of both BRES through the anastomotic strictures and removal of both internal-external drains. **Clinical outcome** : *Success* Major improvement of LFTs. **Follow-up** (22 months): *No relapse* Liver MRI 8 months after stenting: slight dilation of intrahepatic bile ducts confined to the postero-superior right lobe. Improvement in LFTs without need for additional biliary drainage intervention	No procedure-related AEs
**Case 5** 73-year-old female Patient followed for breast tumor in a steady condition. Low-grade dysplasia-IPMN-B located in the left hepatic duct inducing a partial occlusion treated a plastic stent. Decision to perform conservative treatment with radiofrequency ablation. BRES to sustain calibration and prevent further stricturing after RFA.	ERCP confirming a stricture of the left hepatic duct. Single-operator cholangioscopy highlighting a villous intraductal proliferation. Fluoroscopy-guided RFA (Endo-HPB) in a single session, immediately followed by calibration with a bioresorbable 5.7 cm x 10 mm BRES.	**Technical outcome** : *Success* Endoscopic calibration and stricture prevention after radiofrequency. **Clinical outcome** : *Success* No need for unscheduled reintervention during the initial 6 months FU period. Scheduled control ERCP to assess RFA response with cholangioscopy-guided biopsies at 6 months. No visible or microscopic relapse of IPMN-B and neither remaining debris from BRES resorption nor any visible biliary damage, but progression of the cholangioscope beyond the ablated portion required balloon dilation. Scheduled control ERCP with cholangioscopy at 12 months. No RFA-related stricture with easy progression of the cholangioscope. No macro or microscopic relapse of IPMN-B. **Follow-up** (12 months): *No relapse*	No procedure-related AEs
AE, adverse event; BRES, bioresorbable and expandable stent; CT, computed tomography ; ERCP, endoscopic retrograde cholangiopancreatography; EUS, endoscopic ultrasound; FCSEMS, fully-covered self-expandable metallic stent; IPMN, intraductal papillary mucinous neoplasm; IPMN-B, IPMN of the bild duct; LFT, liver function test; RFS, radiofrequency ablation.			

## Discussion

We present what is, to our knowledge, the first case series describing both endoscopic and percutaneous use of magnesium-based expandable BRES in BBS. Our results, despite being subjected to biases of retrospective and small sample studies, remain encouraging with a 100% technical success rate for positioning the stent and an 80% clinical success rate at 12 months for stenting without procedure-related morbidity. Despite inhomogeneous follow-up time, all patients underwent at least 12 months of follow up without stricture relapse among the clinical successes with up to 31 months relapse-free survival.

In the last decade, several bioresorbable stents have been investigated for biliary applications but with moderate efficacy to date, mostly because of poor expansion force, stiffness making insertion difficult, or tissue hyperplasia 8 9 10 . UNITY-B stents have the advantage of being carried by a hydrostatic dilation balloon, allowing calibration of the stricture with a strong radial force and maintaining patency of calibration for up to 3 months. Successful implantation has been reported in a few case series 11 and deployment has been demonstrated by others in free-access videos 12 .

We used UNITY-B BRES to calibrate anastomotic strictures in altered anatomies where a standard FCSEMS stent could not have been subsequently retrieved or would have occluded collaterals, thus avoiding protracted external drainage. All the indications being “benign” at the time of procedure, stenting with unchangeable uncovered SEMSs would have been unwise.

In our patient with an intrahepatic stricture, the only alternative would have been a plastic stent. BRES may have allowed better calibration and prevented early development of a post-RFA stricture. In the patient with a stone formed above a strictured biliodigestive anastomosis, EUS-guided FCSEMS stenting bypassed the stricture and the stone, but BRES allowed consolidation of the neoanastomosis and complete stone clearance with improved intrahepatic biliary drainage. Stenting was performed equally percutaneously or endoscopically with equivalent technical outcomes. All procedures were safely conducted with no infection of other AEs following stent resorption.

In one case, a systematic control cholangioscopy was performed 6 and 12 months after stenting confirming complete stent resorption. In one patient, two stents were seamlessly positioned coaxially to allow for longer calibration of the biliary duct. Because our study was retrospective and followed standard of care, no systematic imaging was scheduled and biliary patency was mostly clinically and biologically assessed. When deployed, the stent is almost invisible under fluoroscopy and positioning relies on radiopaque markers and contrast-filled balloon dilation ( Fig. 1 ). In our only patient with early relapse of the bile duct dilation, a CT was performed at 4 weeks from the initial intervention and no stent remnant was visible. We could not determine whether the stent was still present but obstructed by what appeared later as a tumor relapse, if it had migrated, or if it had dissolved earlier than planned. Stent resorption is an issue with BRES, both to ensure a long enough time of calibration and to prevent local or systemic toxicity. UNITY-B resorption time has been lab-studied but not clinically investigated. There are, however, several safety studies regarding magnesium-based stents underscoring their high biocompatibility 13 14 .

We selected patients in this series to receive BRES for specific reasons of access availability and the expectation of sustained response within 3 months of calibration. However, European Society of Gastrointestinal Endoscopy guidelines for BBS advise longer stenting periods of at least 6 months (FCSEMS) or 12 months 1 . The currently available BRES as used in this study would probably not last long enough to sustain long-term resolution of most BBSs, but a longer-lasting, 6-month version is currently in development and could hold promise for replacing metallic or plastic stenting for benign strictures by avoiding repeat scheduled stent exchanges and removals without limitations regarding risk of obstructing collateral bile ducts.

## Conclusions

In this first series describing percutaneous and endoscopic stenting for BBS with magnesium-based bioresorbable stents, technical success was possible in 100% of cases without AEs and with an 80% rate of sustained clinical success. This work opens the way for larger prospective series evaluating the benefit, safety, and cost-effectiveness of BRESs for management of selected benign biliary strictures, although longer-lasting BRESs are desirable to cover most BBS indications.
